# Compelled Body Weight Shift Technique to Facilitate Rehabilitation of Individuals with Acute Stroke

**DOI:** 10.5402/2012/328018

**Published:** 2012-05-01

**Authors:** Sambit Mohapatra, Aileen C. Eviota, Keir L. Ringquist, Sri Ranjini Muthukrishnan, Alexander S. Aruin

**Affiliations:** 1 Department of Physical Therapy, University of Illinois at Chicago, 1919 West Taylor Street (MC 898), Chicago, IL 60612, USA; 2 Department of Physical Therapy, University of Illinois at Chicago Medical Center, Chicago, IL 60612, USA; 3 Department of Neurology and Rehabilitation Medicine, University of Illinois at Chicago, Chicago, IL 60612, USA

## Abstract

**Background:**

The study evaluates the effectiveness of Compelled Body Weight Shift (CBWS) approach in the rehabilitation of individuals with stroke. CBWS involves a forced shift of body weight towards a person’s affected side by means of a shoe insert that establishes a lift of the nonaffected lower extremity.

**Methods:**

Eleven patients with acute stroke were randomly assigned to experimental and control groups. The experimental group received a two-week conventional physical therapy combined with CBWS and the control group received only a two-week conventional therapy. Weight bearing, Gait velocity, Berg’s Balance, and Fugl-Meyer’s Scores were recorded before and after the intervention.

**Results:**

Weight bearing on the affected side increased in the experimental group and decreased in the control group. The increase in gait velocity with treatment was significant in both the groups (*P* < 0.05). However, experimental group (*P* = 0.01) demonstrated larger improvements in gait velocity compared to the control group (*P* = 0.002). Berg Balance and Fugl-Meyer scores increased for both the groups.

**Conclusion:**

The implementation of a two-week intervention with CBWS resulted in the improvement in weight bearing and gait velocity of individuals with acute stroke. The present preliminary study suggests that CBWS technique could be implemented as an adjunct to conventional rehabilitation program for individuals with acute stroke.

## 1. Introduction

Stroke is the leading cause of serious, long-term disability among American adults. Each year in the United States approximately 795,000 people sustain a new or recurrent stroke [1] and nearly half survive with some level of neurological impairment and disability [2]. It is a common observation that individuals with stroke-related hemiparesis exhibit asymmetry in quasistatic standing postures as well as during functional movements [3–5]. The causes of asymmetries include motor weakness [6], asymmetric muscular tone [7, 8], and somatosensory deficits [6]. Stroke-related asymmetries affect the performance of functional activities. For example, it was reported that asymmetries of stance contribute to balance impairments in individuals with stroke seen as increased postural sway [8], disordered gait, and increased probability of falls [9]. The degree of asymmetrical weight bearing has been correlated with a decrease in motor function, decrease in the level of self-care independence, and increase in the length of hospital stay after stroke [10]. It was also suggested that weight-bearing asymmetry and impaired balance function may be a consequence of learned disuse of the paretic leg [11]. Indeed, it is known that following a stroke, when significant paresis exists, an individual with hemiparesis may be unable or reluctant to bear much weight through the paretic limb. However, a continued weight-bearing asymmetry fosters further disuse of the affected side despite the probability that improved motor function in the lower limb has occurred. Such a learned disuse of the affected limb may contribute to the lack of progress in recovery of some individuals with stroke.

Several treatment approaches are used to improve weight-bearing symmetry in individuals with stroke. Among them are rehabilitation interventions that involve voluntary shifts of body weight based on dynamic visual [12, 13] or auditory feedback [14] about relative weight distribution over the paretic and nonparetic limb, or weight shifting exercises [15]. A single session of using shoe wedges and shoe lifts on the nonaffected side have been shown to improve the symmetry of weight bearing in patients with chronic stroke by compelling them to shift the body weight to the paretic leg [4]. It was also demonstrated that a 10 mm shoe lift is appropriate to induce sufficient symmetry in weight bearing. Such a shoe lift, when coupled with a six-week goal-directed balance exercise, showed significant improvement of walking speed, stride length, and symmetry of weight bearing in a patient with chronic stroke [11]. However, it is not known whether or not individuals with a recent stroke would improve the symmetry of weight bearing while using a shoe lift on the unaffected side. As such, the overall objective of this research was to test the efficacy of a new form of rehabilitation namely, Compelled Body Weight Shift (CBWS) therapy. This rehabilitation therapy involves a forced shift of body weight towards the affected lower extremity by means of a shoe insert that establishes a prolonged lift of the nonaffected lower extremity. The underlying mechanisms of the Compelled Body Weight Shift is that the forced shift of body weight towards the affected lower extremity helps in overcoming the learned disuse of the paretic leg.

We hypothesized that using a shoe lift on the unaffected side combined with conventional physical therapy treatment would improve stance and weight-bearing symmetry in patients with acute stroke. We also hypothesized that patients provided with CBWS therapy, who used a shoe lift on the nonaffected side, would demonstrate greater improvements in gait velocity compared to the patients who were treated with conventional therapy alone.

## 2. Methods

### 2.1. Subjects

The study participants were recruited from the cohort of stroke patients admitted to the University of Illinois at Chicago Medical Center Rehabilitation Unit. The inclusion criteria for the study were a single, acute (less than 20 days), unilateral stroke with asymmetry of weight bearing (weight bearing on the affected side of less than 35% of the total body weight [4, 16]), ability to stand and maintain balance with minimal assist (FIM ambulation score of at least 1), and ability to understand and follow instructions. The exclusion criteria were serious or unstable medical conditions, history of other neurological diseases (i.e., chronic stroke, Parkinson’s disease, and multiple sclerosis), other comorbidities, and fixed contractures or deformity. Eleven individuals who satisfied the inclusion/exclusion criteria were selected. Their mean age was 49.2 ± 3.7 years and the mean time since the stroke accident was 14.7±3.2 days. There were four females and seven males (Table 1). All subjects were ambulatory with assistive devices and had no history of previous strokes. The difference between the groups was not statistically significant: height (*P* = 0.88), weight (*P* = 0.58), age (*P* = 0.19), time since the stroke (*P* = 0.34), and FIM score (*P* = 0.68). The subjects were randomly assigned to two groups: experimental and control. The study protocol was approved by the University of Illinois at Chicago Institutional Review Board. Prior to the experiment, all participants signed a written consent with the objectives and methods of the study clearly explained. The subjects who qualified for the study were selected and recruited over one-year period.

### 2.2. Intervention

**Figure 1:**
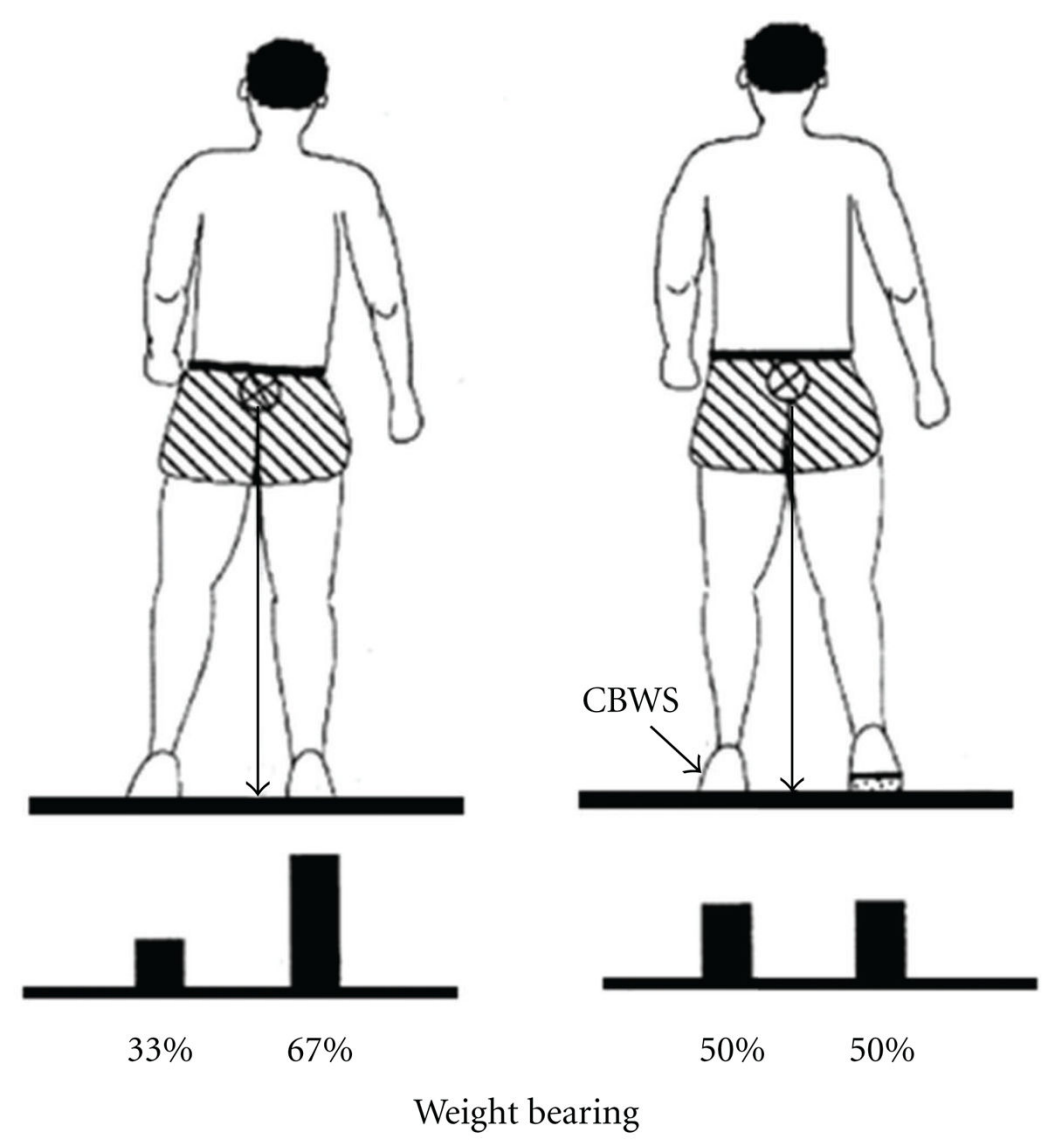
Schematic representation of a stroke-related asymmetry of stance and weight-bearing (left) and how a shoe insert restores weight-bearing symmetry by lifting the nonaffected lower extremity (right).

To achieve the compelled body weight shift, the individuals with hemiparesis included in the experimental group were provided with shoe lifts of 0.6 cm fabricated from medium hardness foam material made of ethylene vinyl acetate, while no shoe lift was provided to those included in the control group. Thus, each subject included in the experimental group wore shoes with the installed shoe lift (innersole) on the unaffected limb during the time of treatment (Figure 1). The subjects in both groups received similar physical therapy treatments, six times a week for two consecutive weeks. The duration of each treatment session was at least 90 minutes on weekdays and 30 minutes on Saturdays. Physical therapy interventions included (a) therapeutic exercises involving active and active-assisted range of motion training, (b) resistive exercises with Thera-Band and/or weights, (c) motor retraining activities such as static and dynamic standing and gait actions, (d) gait training involving walking over ground, on a treadmill, body weight support treadmill training, walking on indoor/outdoor surfaces, and stair training, and (f) functional performance training such as sit to stand maneuvers at varied heights and bed mobility exercises.

### 2.3. Outcome Measures

All participants underwent a battery of identical tests two times, before the start of the rehabilitation intervention (before test) and following its completion (after test).

Weight bearing was measured with a digital weighing scale (Scale-Tronix, 5005 Stand-On Scale). During the assessment, the subject stood in such a way that his/her affected leg was on the platform of the scale while the unaffected leg was on a wide wooden block (with the length 0.51 m, height 0.06 m, and width 0.29 m, which matched the dimensions of the weighing scale platform) adjacent to the platform of the scale. Then, the subject was positioned on the platform of the scale and the entire body weight was recorded. The measurements were repeated three times. Weight bearing on the affected side for each subject was calculated as a percentage of the entire body weight.

Gait velocity was obtained when the subject walked normally with a cane at his/her comfortable walking speed across a 10 m walkway. The time of crossing 5 m along this walkway was recorded with a stopwatch. No lift insert was used during any tests.

Balance performance was determined by the Berg Balance Scale (BBS). The BBS, a 14-item scale designed to measure balance in a clinical setting with a maximum score of 56 [17]. Each item is scored from 0 (cannot perform the task) to 4 (the best performance), including the ability to maintain sitting balance, static and dynamic standing balance, and stability during functional transfer tasks. In this scale, a score of 0–20 indicates that the subject is wheelchair bound, a score of 21–40 suggests the individual needs assistance while walking, and a score of 41–56 means the individual is independent [18]. This test has been shown to be correlated with other tests of mobility and balance, including the Tinetti mobility index and the Get Up and Go tests [17], and it is considered to be a valid and reliable clinical tool [19].

The Fugl-Meyer Assessment (FMA) [20] for the lower extremities was administered for the following five domains (total score = 100): motor function (maximum score = 34), sensation (maximum = 12), sitting/standing balance (maximum = 14), joint range (maximum = 20), and joint pain (maximum = 20). This test has been shown to be valid and reliable for assessing the recovery of function [21], is correlated with the capacity to perform ADL activities [20], and is commonly used for measuring motor recovery after stroke [22].

### 2.4. Data Analysis

Descriptive analysis was used to analyze the demographic data in each subject group. Split plot ANOVAs were performed with time (before test and after test) as the within-subjects factor for the various dependent variables (weight bearing, gait velocity, BBS, and FMA) whereas group (experimental or control) was the between-subjects factor. SPSS 17 software for Windows 7 was used for data analysis (SPSS Inc., Chicago, IL, USA). For all tests, statistical significance was set at *P* < 0.05.

## 3. Results

**Figure 2:**
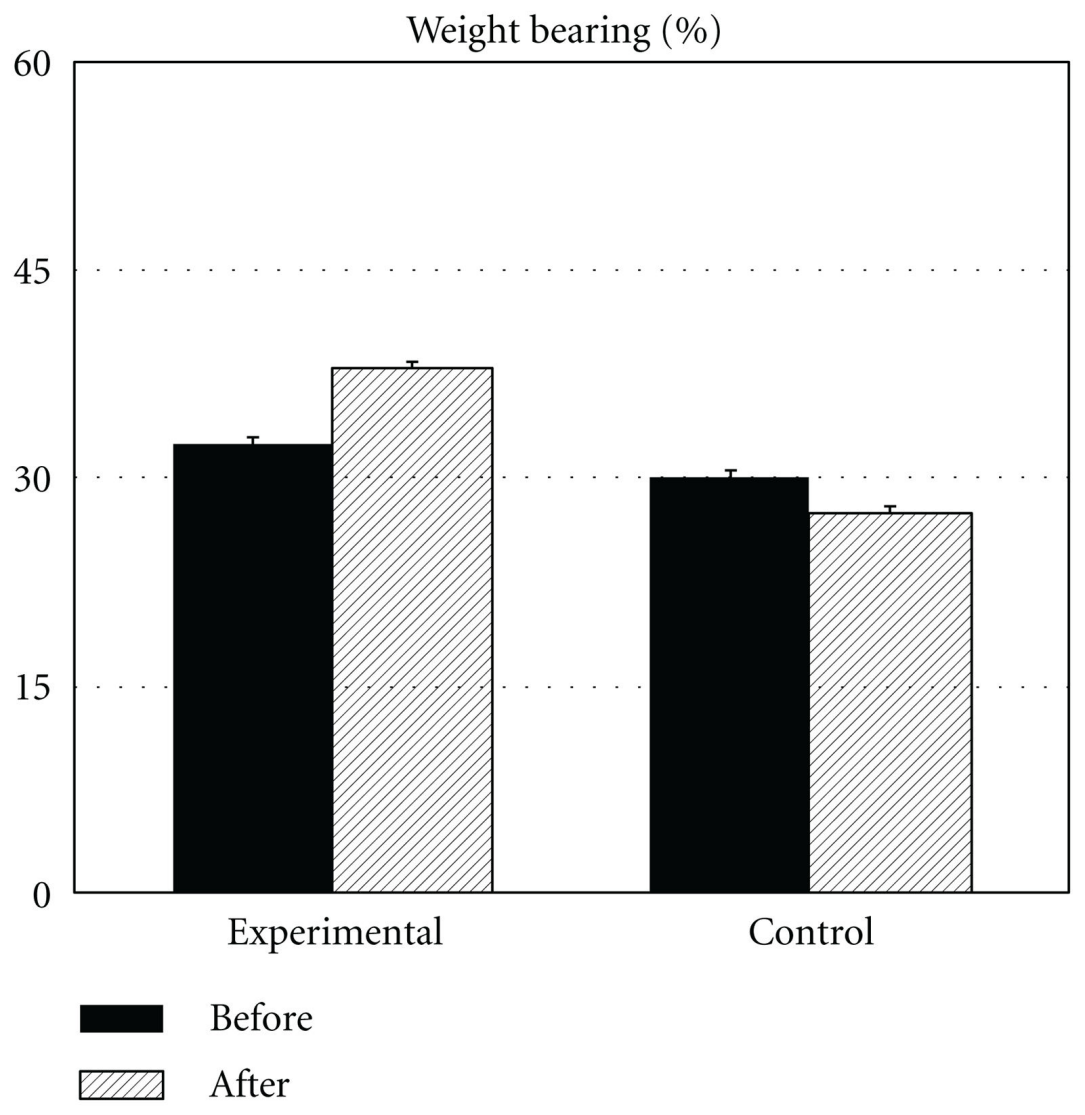
Changes in the percentage of weight bearing on the affected side (% of the total body weight). Mean ± SE are shown.

Figure 2 shows the mean group data for weight bearing obtained before the start of intervention and after the end of the intervention. All of the subjects demonstrated asymmetrical weight bearing at the time of the first test: weight bearing of the individuals included in the experimental group was 32.4 ± 0.06 and it was 30.2 ± 0.04 percent of the body weight in the control group. The difference between the experimental and control subjects was not statistically significant (*P* = 0.89). The mean weight bearing increased after test reaching 37.9 ± 0.05 percent in the experimental group of subjects approaching the level of statistical significance (*P* = 0.07). The mean weight bearing on the affected side of the subjects in the control group decreased after test to 27.4 ± 0.06; however, such a decrease was not statistically significant (*P* = 0.29). The difference between the groups was not statistically significant (*P* = 0.44).

**Figure 3:**
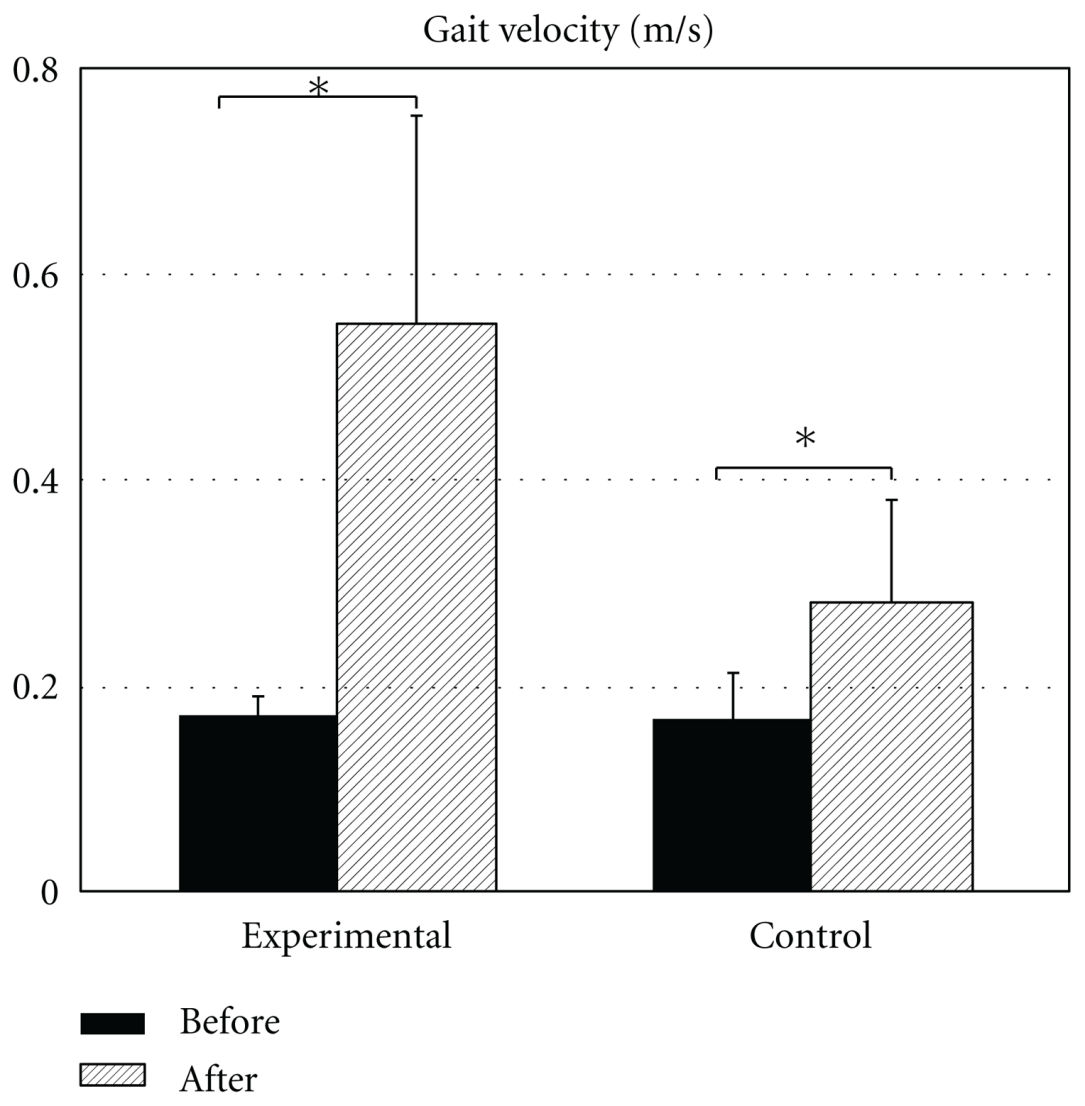
Changes in gait velocity (in m/s) with treatment. Mean ± SE are shown. ^*^shows statistical significance at *P* < 0.05.

Figure 3 shows the mean group data for gait velocity. Before the start of treatment, individuals included in the experimental group and those included in the control group demonstrated similar gait velocities of 0.17 ±0.02 and 0.17 ± 0.04 m/s, respectively (*P* = 0.66). After intervention, gait velocity increased in both groups reaching 0.55 ± 0.2 m/s in the experimental and 0.28 ± 0.1 m/s in the control groups. While the increase in gait velocity with treatment was significant in both the experimental (*P* = 0.01) and control (*P* = 0.002) groups, the experimental group showed greater improvements in gait velocity than the controls, although the difference was not statistically significant (*P* = 0.51).

Before the start of treatment, the BBS of the individuals included in the experimental group was 19.2 ± 3.1 and it was 13.2 ± 3.06 in the control group: the scores were not significantly different between the groups (*P* = 0.57). After completion of the two-week intervention, BBS improved reaching 41.2 ±1.9 in the experimental group and 36.7 ±2.4 in the control group. ANOVA revealed the effect of treatment for the experimental (*P* = 0.003) and control groups (*P* = 0.001). However, the difference between the groups was not statistically significant (*P* = 0.46).

Before the start of the intervention, the total FMA score (lower extremities) for the experimental group was 77 ± 1.7 and the control group had a score of 73.3 ± 3.5. This difference in FMA scores between the experimental and the control groups was not statistically significant (*P* = 0.66). After test, FMA scores improved for both groups reaching 89 ± 0.9 for the experimental and 86 ± 4.0 for the control groups (*P* = 0.67). The results of ANOVA (*P* = 0.003) and (*P* = 0.001) for the experimental and control groups, respectively, confirmed the main effect of the time.

## 4. Discussion

There is a consensus among clinicians regarding the importance of retraining the ability of individuals suffering from stroke to maintain symmetrical stance [12]. Accordingly, there is a need to develop simple and efficient rehabilitation approaches to restore symmetrical stance after stroke. One such novel approach, CBWS therapy, was evaluated in the current study. The main finding was the improvement of symmetry of weight bearing with intervention involving a CBWS, the compelled shift of body weight towards the subject’s affected side. The study outcome supports the first hypothesis that implementing a shoe lift on the unaffected side during conventional physical therapy improves symmetry of stance and weight bearing of the paretic lower extremity. The second hypothesis was also supported as the patients provided with the shoe lift demonstrated larger improvements in gait velocity compared to the patients who were treated using only conventional therapy.

### 4.1. Role of CBWS in the Improvement of Symmetry of Weight Bearing

The results of the current study demonstrated that individuals with acute stroke improve the symmetry of weight bearing while participating in conventional physical therapy combined with CBWS therapy. Why were the patients who were provided with a shoe lift able to learn to transfer more weight to the paretic leg than the control group who was not given the lift? One possible explanation is that a simple shoe lift would compel the patient to shift more weight to the affected side. Such a compelled redistribution of body weight resembles the concept of “force use” of the affected extremity as promoted by Taub et al. [23]. The improvement of weight-bearing symmetry while using a shoe lift most likely helped those individuals avoid the development of learned disuse of the affected limb. It is important to note that individuals in the control group did not show improvement in weight-bearing symmetry and instead showed minor worsening after treatment. As such, the observed decline in weight-bearing symmetry suggests that individuals in the control group could develop learned disuse of the affected limb and asymmetrical stance and would require additional treatment to eliminate such a probability.

Previous studies suggest that the impaired ability to shift weight onto the nonparetic leg is more pronounced in patients with right-cortical damage [24]. As such, it is not surprising that subjects in the experimental group who had left side damage showed 8.7 ± 3.9 percent improvement of weight bearing on the affected side, whereas patients with right hemispheric lesions showed only 0.89 ± 0.5 percent improvement in weight bearing. Such differences in the achievement of more symmetrical weight bearing between the patients with right and left hemispheric lesions suggests for a need to tailor the CBWS protocol to treatment of individuals with right hemispheric lesions as they might need more time to improve weight-bearing symmetry compared to the patients with left hemispheric lesions. This study outcome however, could be considered only as preliminary because the difference in the percentage of the weight bearing improvement was not statistically significant due to the small subgroup sizes.

It was shown previously that individuals with chronic stroke provided with a shoe lift show improvements in symmetry of quite stance [4, 11]. Similarly, individuals postacute stroke, trained with the feedback device (that provided dynamic visual information about relative weight distribution over the paretic and nonparetic limb) showed significantly better static standing symmetry than subjects who did not receive augmented feedback [13]. It was also reported that a training program that was based on weight transfer and balance exercises performed under different conditions of manipulation of sensory inputs resulted in a significant improvement in the ability to maintain balance control in patients with chronic stroke [25]. Furthermore, an increase in weight bearing on the paretic leg (from 41–42 percent to 65–68 percent) has been reported in post-acute patients with hemiparesis when they placed their nonparetic foot on a step, regardless of step height (10 cm or 17 cm) [26]. It is important to note that the literature data suggests that individuals following stroke are able to bear more than 50 percent of their body weight through the affected lower extremity [16]. As such, the difficulties in restoring the symmetry of weight bearing that many individuals with stroke experience are due to a learned disuse of the affected leg rather than the impaired ability of the affected leg to bear the weight of the body.

### 4.2. Role of CBWS in Improvement of Gait Velocity

The limited walking ability that follows a stroke significantly limits the patients’ capability to participate in many community activities. Moreover, gait velocity has been reported to be a predictor of the severity of impairment [27] and the restoration of the ability to walk is considered to be the major goal of stroke rehabilitation [28]. Most common methods used to restore gait in individuals suffering from a stroke include functional electrical stimulation (FES) [29], body weight-supported treadmill training (BWSTT) [28], and robotic-assisted gaitretraining [30]. While the importance of the above-mentioned approaches is acknowledged, other methods are used which do not require expensive equipment and which can be implemented in any clinical facility. Such methods include overground walking combined with a traditional functional strengthening exercise and practicing single movements or various neurofacilitation techniques [28]. Moreover, it was reported in the literature that overground walking enhances locomotor recovery more than other forms of therapy [31].

The outcome of the current study demonstrated that gait velocity is improved when individuals with stroke use a shoe lift on the nonaffected side during treatment. It is important to note that the improvement of gait velocity was achieved in parallel with the improvement in weight-bearing symmetry. This result is in contrast with the previously published data on the lack of observed improvement of walking function after the use of a feedback device that provided visual information about relative weight distribution over the paretic and nonparetic limb [13]. The improvement in gait velocity in our study could be associated with the fact that weight-bearing symmetry was achieved by providing a shoe lift during the entire time of treatment that involved ambulation. Such a combination of interventions (overground gait and a compelled shift of the body weight) had a positive effect on the ability of a patient to overcome (or to prevent the development of) a learned disuse of the affected leg.

It is also important to note that while both groups of subjects increased the velocity of their gait with treatment, individuals in the experimental group showed a 30 percent increase in the velocity of their gait while the control subjects demonstrated only a 6 percent increase in gait velocity. Thus, using the CBWS as an addition to conventional physical therapy resulted in the ability of the patients in the experimental group to achieve gait velocity that is considered at the level of limited community ambulation whereas in the control group the gait velocity increased only to the level of ambulation within the household [32].

### 4.3. Role of CBWS in Improvement of Clinical Measures

The restoration of balance and the enhancement of motor recovery continue to be major rehabilitation goals for persons with stroke. Both groups showed improvement in their BBS and FMA after treatment. However, the individuals in the experimental group showed larger improvements in both the BBS and FMA compared to the control group. Thus, the BBS increased in individuals receiving CBWS by 22 points (46 percent) while in the control group it increased by 23.5 points (35 percent). It was also demonstrated that a difference of five to seven BBS points is necessary to conclude with 90 percent certainty that patients receiving rehabilitation following stroke have undergone a real change in BBS when assessed in a between-rater situation [33]. Thus, we can conclude that a 46 percent increase in BBS is certainly a manifestation of the effect of the CBWS approach. Moreover, individuals in the experimental group demonstrated BBS that are closer to that proposed in the literature which was a 45–58 point cut-offvalue for predicting a high risk for falls associated with clinically impaired balance and transfer ability in healthy elderly subjects [34]. However, this increase in BBS in the experimental group should be considered with caution because the groups had slightly different initial scores (although not statistically significant), which most likely masked the effect of using the shoe lift in the improvement of BBS.

The FMA score for lower extremity has been a moderate predictor of both improvements in gait velocity and stride length in patients with stroke [35, 36]. In our study, the FMA scores in the experimental group increased by 12 points while the subjects in the control group increased the scores by 12.7. Relatively similar FMA gains in both of the groups could be explained by the short duration of the intervention because it is expected that motor recovery continues after the initial two-week period. As such, future studies are needed to assess the effect of CBWS therapy on motor recovery.

There are several limitations that should be considered. First, the findings of this study could only be applied to the relatively young, acute patients who are able to walk without physical assistance for 10 m. The effectiveness of CBWS on patients with higher levels of impairment is not clear. Second, many of the contacted patients who expressed an interest were unable to walk without physical assistance as such the number of participating subjects was relatively small. Third, the effect of different parameters of CBWS such as duration of treatment and its intensity, height of the lift, and so forth were not examined in the present study. Fourth, the long-term effect of CWBS was not examined in this research. As such, a larger study is needed to investigate the effect of CBWS on acute stroke patients with different mobility levels, the parameters and intensity of CBWS, and the carry-over effects of CBWS.

## 5. Conclusions

A two-week intervention involving compelled body weight shift therapy induced by a shoe lift on the unaffected side led to an improvement in the symmetry of weight bearing and gait velocity in individuals with acute stroke. Thus, a new technique helps to facilitate rehabilitation of individuals with acute stroke.

## Figures and Tables

**Table 1:** Descriptive characteristics of study participants (*n* = 11).

Subject no.	Height (m)	Weight (kg)	Age, years	Gender	Ischemic (I)/Hemorrhag (H)	Location of stroke	Time since stroke (days)	FIM ambulation score at start of treatment
Experimental group								

(1)	1.8	83.5	42	Male	I	Right temporo parietal	12	1
(2)	1.6	70.3	59	Female	H	Left thalamic	10	1
(3)	1.8	102.5	33	Male	I	Right parieto-occipital	13	2
(4)	1.6	54.9	49	Male	H	Left frontoparietal	8	2
(5)	1.6	81.6	36	Female	I	Right middle cerebral artery (MCA)	13	4

Mean ± SE	1.68 ± 0.05	78.56 ± 7.9	43.8 ± 4.7				11 ± 0.9	2 ± 0.5

Control group								

(6)	1.9	94.8	41	Male	H	Left posterior temporal and parieto-occipital	7	4
(7)	1.8	74.4	52	Male	I	Right MCA occlusion	9	2
(8)	1.5	52.6	62	Female	H	Left intraparenchymal and subarachnoid	21	2
(9)	1.5	72.6	73	Female	H	Left thalamic	11	2
(10)	1.7	83.9	40	Male	H	Right basal ganglia and temporal	45	2
(11)	1.6	59	54	Male	I	Right embolic	13	1

Mean ± SE	1.66 ± 0.07	72.88 ± 6.3	53.7 ± 5.1				18 ± 6	2.2 ± 0.4

FIM: Functional Independence Measure. The difference between the groups was not statistically significant: height (*P* = 0.88), weight (*P* = 0.58), age (*P* = 0.19), time since the stroke (*P* = 0.34), and FIM (*P* = 0.68).
